# Patterns and prognostic factors of metastasis in Latino/Hispanic patients with melanoma and negative lymph nodes

**DOI:** 10.3332/ecancer.2025.1905

**Published:** 2025-05-13

**Authors:** Jessica J Farzan, Jiddu Guart, Gabriel De la Cruz Ku, Nichita Kulkarni, Rachel Huselid, Anshumi Desai, Camila Franco, Vanessa Mroueh, Jessica Mroueh, Gonzalo Ziegler

**Affiliations:** 1 University of Massachusetts Medical School, Worcester, MA 01655, USA; 2 Universidad Cientifica del Sur, Lima 15067, Peru; 3 Division of Plastic and Reconstructive Surgery, DeWitt Daughtry Family Department of Surgery, University of Miami Miller School of Medicine, Miami, FL 33136, USA; 4 Department of Surgery, University of Texas Medical Branch at Galveston, Galveston, TX 77555, USA; 5 Department of Plastic Surgery, Brigham and Women’s Hospital, Harvard Medical School, Boston, MA 02115, USA; 6 Department of Plastic Surgery, University of Pittsburgh Medical Center, Pittsburgh, PA 15219, USA; 7 Department of Breast, Melanoma, and Sarcoma Surgical Oncology, Instituto Nacional de Enfermedades Neoplasicas (INEN), Lima 15038, Peru; 8 Chief, Cutaneous Oncology Unit, Clinica Ziegler, Lima 15036, Peru

**Keywords:** melanoma, sentinel lymph node biopsy, Hispanic/Latino

## Abstract

**Introduction:**

Malignant melanoma presents with diverse clinical and histological manifestations that vary per population. Lymph node status, assessed through sentinel lymph node biopsy, is a widely accepted standard of care and a key prognostic indicator. This study aims to identify clinical outcomes, clinicopathologic factors, recurrence patterns, metastatic spread patterns and risk factors associated with lymph node-negative melanoma in our Latino/Hispanic patient population.

**Methods:**

We included patients diagnosed with lymph node-negative melanoma at the Instituto Nacional de Enfermedades Neoplásicas, Lima, Peru, from 2010 to 2019. Cox regression analysis was used to assess prognostic factors.

**Results:**

The study included 249 patients with lymph node-negative melanoma, with a median follow-up time of 25 months. Recurrence was observed in 27% of patients, with a mean age of 65 years compared to 60 years in the non-recurrent group. With a median follow-up of 35 months, the 3-year recurrence-free survival (RFS) rates and overall survival (OS) were 75% and 94%, respectively. The 3-year OS rate was 97% and 88% for non-recurrent and recurrent patients, respectively (*p* = 0.002). The predictors of RFS were Breslow index (hazard ratio (HR) = 1.098, 95%CI: 1.051–1.146, *p* < 0.001) and number of mitoses per mm^2^ (HR = 2.105, 95%CI: 1.150–3.852, *p* = 0.016). Age was the only predictor for lymph node recurrence (HR = 1.053, 95%CI: 1.010–1.098, *p* = 0.016), and Breslow index for distant metastasis (HR = 1.126, 95%CI:1.059–1.196, *p* < 0.001). Breslow index was the only prognostic factor for OS (HR = 1.090, 95%CI:1.034–1.150, *p* = 0.001).

**Conclusion:**

The Latino/Hispanic population has unique characteristics and prognostic factors for oncologic outcomes. Increased Breslow depth and number of mitoses per mm^2^ were significant predictors of recurrence in lymph node-negative melanoma. There is a need for personalised risk assessment and management strategies in this population in terms of surveillance and adjuvant therapies. Further molecular and genetic predictors and markers of recurrence need to be investigated.

## Introduction

Melanoma, a malignant neoplasm originating from melanocytes, exhibits a diverse array of clinical and histological manifestations. A pivotal prognostic indicator is lymph node status, assessed through sentinel lymph node biopsy (SLNB), a widely accepted standard of care [1]. Melanoma with positive sentinel lymph nodes (SLNs) is a significant prognostic factor, alongside Breslow thickness and the presence of ulceration [2].

The integration of SLNB into the standard of care has facilitated more accurate staging and risk stratification, guiding personalised treatment strategies and surveillance protocols for melanoma patients. Indications for SLNB encompass primary melanomas characterised by ulceration, Breslow depth exceeding 1 mm or meeting the criteria for Clark level IV or V [1]. Furthermore, SLNB is considered on a case-by-case basis for lesions with a Breslow depth ranging from 0.8 to 1 mm or less than 0.8 mm with concomitant ulceration [3]. SLNB is also recommended for patients presenting with intermediate-thickness melanomas, defined as Breslow depth between 1 and 4 mm, as well as thick melanomas, with a Breslow depth exceeding 4 mm. However, for thick melanomas, the prognostic significance of a positive lymph node may be less pronounced, as these patients are already classified as high-risk [3].

Patients with lymph node-negative melanoma tend to exhibit a more favourable prognosis compared to those with lymph node-positive disease [4]. However, despite negative lymph node involvement, a subset of patients still develop distant metastases, complicating their clinical outcomes and underscoring the complexity of melanoma progression [4].

This study aims to identify the clinical outcomes, clinicopathologic factors, patterns of recurrence, metastatic spread patterns and specific risk factors associated with lymph node-negative melanoma in the Latino/Hispanic patient population. A comprehensive analysis of these factors will enhance prognostication capabilities and lead to better risk stratification. As a result, treatment approaches can become more personalised, optimising clinical management, improving overall survival (OS) and enhancing the quality of life for this population.

## Materials and methods

### Study design

A retrospective cohort study of patients who were diagnosed with melanoma at the Instituto Nacional de Enfermedades Neoplásicas (INEN), Lima, Peru, from 2010 to 2019 was conducted. The data were obtained from medical records. The inclusion criteria were: (1) patients with negative lymph nodes confirmed by pathology review, (2) only stage I and II melanoma, (3) confirmed diagnosis of melanoma by pathology review, (4) anatomic areas such as trunk and extremities and (5) all specimens reviewed in the pathology service at INEN. Exclusion criteria included mucosal melanoma, primary lesions located in the head and neck, lymph nodes positive for metastasis, recurrent melanoma at diagnosis, patients treated at another institution and incomplete medical records.

### Variables

The eighth edition of the American Joint Committee on Cancer (AJCC) Staging Manual was used to classify the primary tumour and initial lymph node status [5]. Sociodemographic, clinical, pathological and surgical variables were included. Patients were stratified by the presence or absence of recurrence, as well as subgroup analysis according to Breslow depth. Metropolitan and rural status definitions were based on the United States Department of Agriculture, metropolitan areas were areas with central counties with one or more urban zones with populations of 50,000 or more. Rural status was defined as residing in non-metropolitan areas with difficult access to oncological centers.

Recurrence was defined as the regrowth of melanoma close to where the primary tumour was excised or lymph nodes positive for metastatic melanoma confirmed by pathology after the initial surgical intervention. Recurrence-free survival (RFS) was defined as the interval between the time of primary surgical intervention and the date of recurrence confirmed by biopsy pathology. The time between the initial surgical intervention and the date of recurrence was at least 90 days, if the recurrence occurred before this interval, this was considered as metastatic at diagnosis. OS was defined as the interval between the time of diagnosis of melanoma and death by any cause or the end of the study. All patients were treated according to the National Comprehensive Cancer Network (NCCN) guidelines at a comprehensive cancer center [5]. Patients without metastasis at diagnosis underwent local excision with SLNB. Surveillance of the patients was conducted according to the NCCN guidelines, followed every 3 months for 2 years, then every 6 months for 3 years and then annually according to clinical status. Imaging studies were performed as needed during the follow-up. Patients did not receive chemotherapy. Immunotherapy, neoadjuvant chemotherapy was not administered to any patient nor involved in clinical trials. The only chemotherapy regimen administered to the selected patients after the multidisciplinary decision was dacarbazine. Checkpoint inhibitors and targeted therapies are not available in public hospitals in Peru.

### Data analysis

Descriptive statistics were used to assess the sociodemographic, clinical, pathological and surgical characteristics. The chi-square test was used to assess the relationship between categorical variables, while the *t*-student test, Mann Whitney-*U* test and ANOVA test were used to assess the relationship between categorical and quantitative variables, with normal, abnormal distribution and more than three categories, respectively. Prognostic factors for RFS, lymph node RFS, distant metastasis-free survival and OS were assessed with univariate and multivariate Cox regression analysis. A *p* value < 0.05 was considered as statistically significant. We used the Statistical Package for Social Sciences software (version 28.0).

### Ethics

This study was approved by the Institutional Review Board of the INEN. Variables that could have identifiable information about the patients were not reported. Patients and their related clinical information were codified in a database.

## Results

Of the 249 patients, 67 (26.9%) experienced recurrence, while 182 (73.1%) did not. The mean age of patients with recurrence was significantly higher at 65.91 years compared to 60.64 years for those without recurrence (*p* = 0.017). Patients aged 65 years and older had a higher recurrence rate (58.2%) compared to those younger than 65 years (32.8%, *p* = 0.005). Gender, residence and anatomic site did not significantly differ between patients with and without recurrence. Notably, a higher proportion of patients with recurrence had ulcers (78.8% versus 56.1%, *p* = 0.001) and a greater Breslow thickness (mean 7.42 versus 40.65 mm, *p* = 0.002). The number of mitoses per mm^2^ was also significantly associated with recurrence, with 71.4% of patients with recurrence having more than 2 mitoses compared to 47.6% of those without recurrence (*p* = 0.001). AJCC stage II was more common among patients with recurrence (91.0%) compared to those without (68.7%, *p* < 0.001). Adjuvant chemotherapy was more frequently administered to patients with recurrence (13.6% versus 3.8%, *p* = 0.006) Table 1.

The most common sites of recurrence were the lung (10.0%), skin and subcutaneous tissue (9.2%) and solid organs (12.9%). Lymph node recurrence occurred in 8.4% of patients, while the brain, liver, bone and gastrointestinal tract, were less common. 8.4% of patients presented with multiple metastasis and 12.9% with solid organs Table 2.

Stratified analysis by the Breslow index showed that when the index was greater than 4 mm, they had a higher mean age (65.08 years) compared to those with Breslow ≤1 mm (56.97 years, *p* = 0.014). Ulceration was significantly associated with greater Breslow index, with 83.7% of patients with Breslow >4 mm having ulceration compared to 14.7% with Breslow ≤1 mm (*p* < 0.001). Higher Breslow index was also associated with increased recurrence rates (*p* < 0.001). The number of mitoses per mm^2^ was higher in patients with greater Breslow index, with 73.7% of patients with Breslow >4 mm having more than 2 mitoses (*p* < 0.001). AJCC stage II was predominant in patients with higher Breslow index (100% for >4 mm) compared to those with lower Breslow index (25.0% for 1.01–4.00 mm, *p* < 0.001) Table 3.

Further analysis according to the site of metastasis showed that stage II compared to stage I had a higher frequency of metastasis to multiple organs (95.2% versus 4.8%, *p* = 0.024). Stage IB had a tendency to have metastasis to the lung compared to stage IA (100% versus 0%, *p* = 0.051). There was no relation between sub stage II and the site of metastasis. While patients with more than 2 mitosis per mm^2^ had a higher frequency of metastasising to solid organs (71.0% versus 29.0%, *p* = 0.043) and tended to have the brain as the main organ (81.8% versus 18.2%, *p* = 0.059) Table 4.

In multivariate Cox regression analysis, the Breslow index was a prognostic factor for the RFS hazard ratio (HR 1.098, 95% CI 1.051–1.146, *p* < 0.001), as well as the number of mitoses per mm^2^ (HR 2.105, 95% CI 1.150–3.852, *p* = 0.016) Table 5. Moreover, only age was found to be a significant predictor in multivariate analysis for lymph node recurrence (HR 1.053, 95% CI 1.010–1.098, *p* = 0.016) Table 6. For distant metastasis, multivariate analysis showed that the Breslow index was a significant predictor (HR 1.126, 95% CI 1.059–1.196, *p* < 0.001) Table 7. Similarly, the Breslow index was found to be a prognostic factor for worse OS (HR 1.090, 95% CI 1.034–1.150, *p* = 0.001) Table 8. Overall, Breslow thickness emerged as a consistent predictor across various analyses, highlighting its significance in RFS, distant metastasis and OS among Latino/Hispanic patients with melanoma and negative lymph nodes.

**Figure 1.**
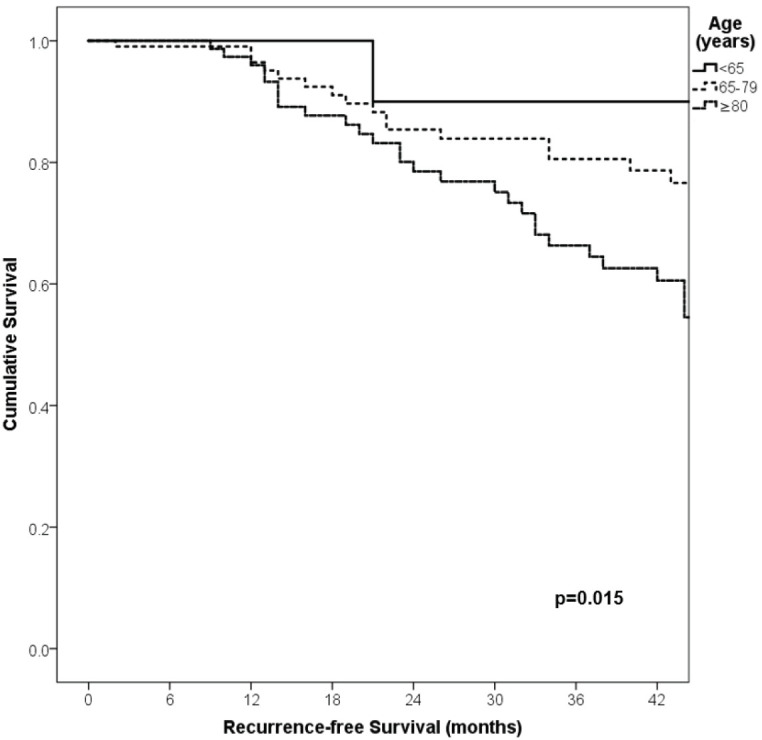
Comparison of RFS from Latino/Hispanic patients with melanoma and negative SLNs according to age.

**Figure 2.**
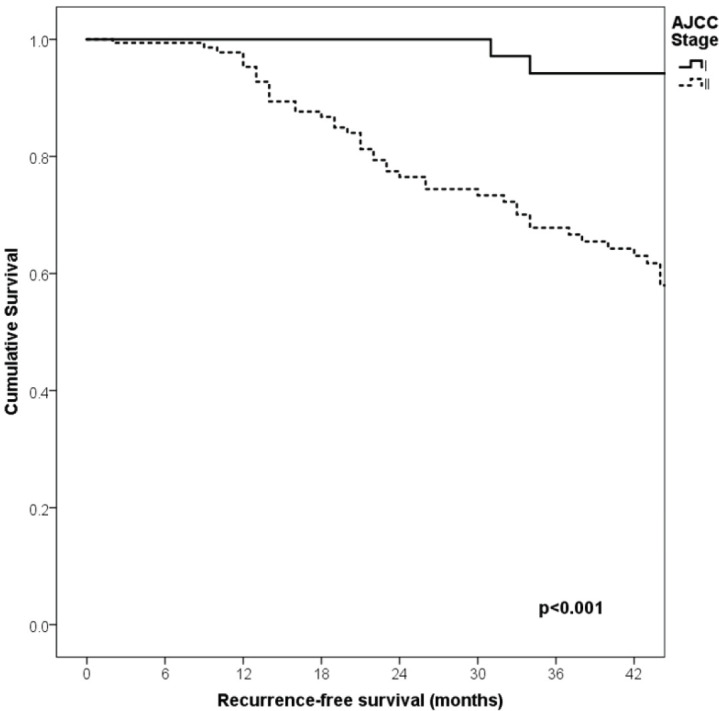
Comparison of RFS from Latino/Hispanic patients with melanoma and negative SLNs according to AJCC stage.

**Figure 3.**
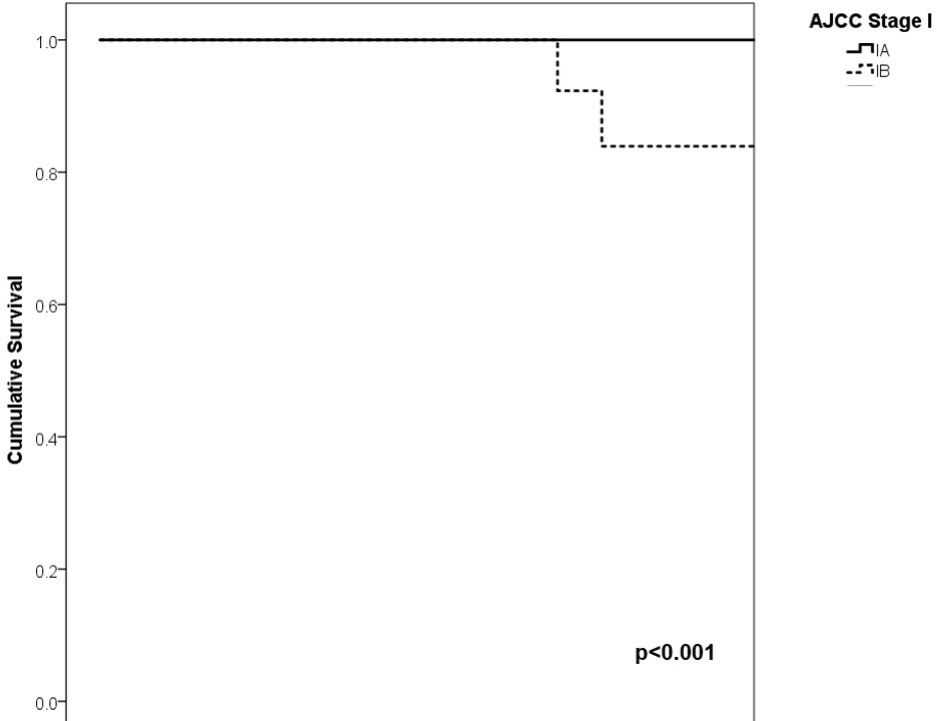
Comparison of RFS from Latino/Hispanic patients with melanoma and negative SLNs according to subgroup of AJCC stage I.

**Figure 4.**
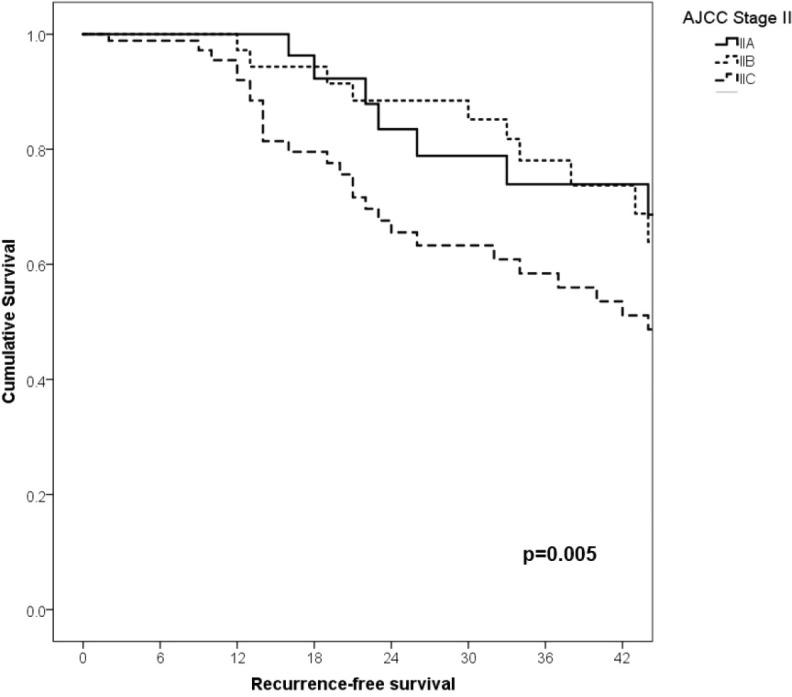
Comparison of RFS from Latino/Hispanic patients with melanoma and negative SLNs according to subgroup of AJCC stage II.

**Figure 5.**
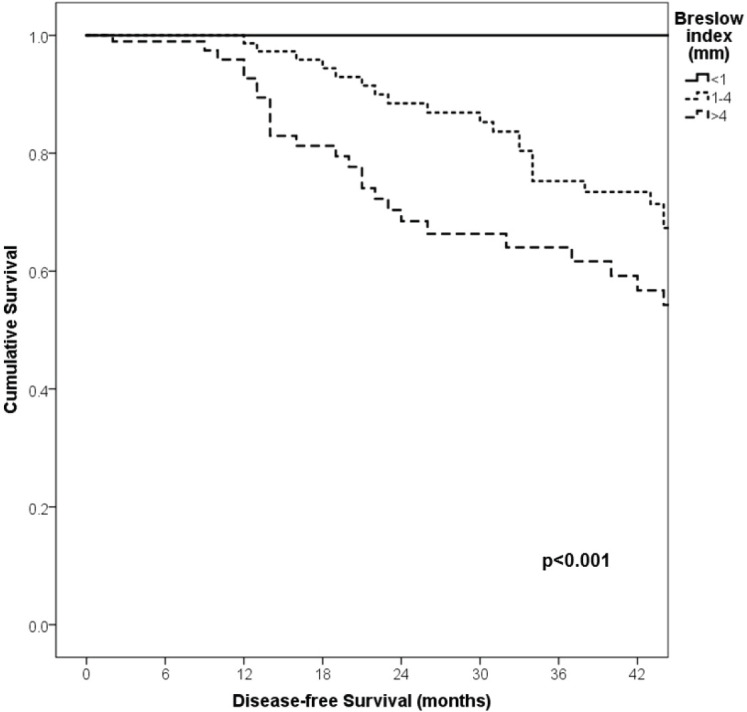
Comparison of RFS from Latino/Hispanic patients with melanoma and negative SLNs according to Breslow index.

With a median follow-up of 35 months, the 3-year RFS rates and OS were 75% and 94%, respectively. In terms of RFS, patients worse outcomes were those with 80 years old or more compared to younger patients 65–79 years and less than 65 years (3 years RFS, 90%, 66%, 81%, *p* = 0.015, respectively), higher AJCC stages (II versus I, 68% versus 94%, *p* < 0.001), higher Breslow index (>4 versus 1–4 mm versus <1 mm; 100% versus 75% versus 64%, *p* < 0.001), presence of ulcer (64% versus 91%, *p* < 0.001), higher number of mitosis per mm^2^ (>2 versus 0–2, 63% versus 88%, *p* < 0.001) Table 9, Figures 1–5. Moreover, when OS survival was assessed at 3-year OS, higher AJCC stages (II versus I;

91% versus 100%; *p* = 0.021), higher Breslow index (>4 versus 1–4 mm versus <1 mm; 85% versus 98% versus 100%; *p* = 0.006) and number of mitosis per mm^2^ (>2 versus 0–2; 91% versus 91%, *p* = 0.026) were associated with worse rates. Furthermore, the 3-year OS rate was 97% and 88% for non-recurrent and recurrent patients, respectively (*p* = 0.002) Table 9, Figures 1–10.

## Discussion

This study investigated characteristics and prognostic factors in Latino/Hispanic patients with lymph node-negative melanoma (stages I and II). While patients with lymph node-negative disease generally have a better prognosis compared to those with lymph node-positive disease, they remain at risk for metastasis and unfavourable outcomes [4]. By understanding the factors associated with lymph node-negative melanoma in this specific population, we aim to identify patients at higher risk for recurrence and tailor their management accordingly.

Carr *et al* [6] reported that 88% of SLNB are negative. Though SLNB are helpful for early detection of disease spread, they are not infallible. Melanoma can recur in the lymph nodes after an initially negative SLNB result, a phenomenon known as a false-negative SLNB [7, 8]. Our study’s false-negative lymph node rate of 8.4% is consistent with other research reporting false-negative rates ranging from 5.6% to 21% [9]. Higher rates of false-negative SLNB have been linked to several risk factors, including older age, deeper lesions, ulceration and head and neck location [7, 9]. A negative SLNB in patients with disease meeting these criteria should be interpreted with caution; as a completion lymphadenectomy could decrease chances of recurrence and improve OS outcomes [10].

In our study, recurrence in lymph node-negative melanoma was significantly related to Breslow depth, older age, mitosis per mm^2^ and AJCC stage II. These findings align with established risk factors in the general population, suggesting that these prognostic indicators may be applicable across diverse ethnic groups. Recurrence did not differ significantly by gender, primary lesion site or histologic subtype. It is well recognised that males have a higher incidence of melanoma compared to females [11–14]; however, more females were included in our study, and we did not observe a significant difference in recurrence rates between males and females with lymph-node negative disease.

While acral lentiginous melanoma and nodular melanoma were the most common histologic subtypes among our participants, recurrence did not differ significantly between groups. This contrasts with some reports linking nodular melanoma to increased lymph node positivity and poorer outcomes, potentially due to its tendency to lack a radial growth phase [1]. Our findings suggest that histologic subtype may not be a major prognostic factor for recurrence in lymph node-negative Latino/Hispanic patients, highlighting the need for further research into potential ethnic-specific variations in melanoma behaviour.

A little less than half of our patients with lymph node-negative disease presented with Breslow thickness greater than 4 mm, and of these patients, approximately 66% did not experience recurrence. This observation suggests that while Breslow thickness is traditionally considered a strong predictor of lymph node status and recurrence [1], other factors may also significantly influence outcomes in Latino/Hispanic patients with thick melanomas. It indicates that the role of Breslow thickness might be more complex than previously understood, and additional variables could be important in determining patient prognosis such as age, number of mitosis per mm^2^ and immunohistochemical and genetic factors not able to be assessed in our manuscript.

Our findings show that RFS is primarily influenced by the Breslow index and mitosis per mm^2^. While White *et al* [15] identified ulceration and tumour thickness as important predictors of survival outcomes, they concluded that the number of positive lymph nodes is the most significant prognostic factor. This suggests that, regardless of lymph node status, ulceration and tumour depth are reliable factors for predicting RFS, which for Latino/Hispanic population we concluded that these prognostic factors are different.

We did not find a significant association between the anatomic location of the primary lesion and RFS in our cohort, which contrasts with some previous studies. For example, Cadili and Dabbs [1] found that primary tumour location was associated with nodal status, with head and neck tumours less likely to have the node-positive disease. This discrepancy between our findings on RFS and previous research on nodal status warrants further investigation. It could be due to the limited statistical power of our study or may suggest that the influence of anatomic location on outcomes varies between lymph node-negative and lymph node-positive patients. Additionally, there might be differences in melanoma behaviour among Latino/Hispanic patients compared to other populations.

In regards to lymph node recurrence, age 65 or older was identified as the only significant predictor of lymph node recurrence in our lymph node-negative cohort. This finding aligns with existing research that shows older adults with melanoma often present with more advanced disease and experience poorer outcomes, regardless of nodal status [12, 13]. This is particularly important because older patients are at a higher risk of false-negative results in lymph node evaluations. Given these risks, there is a clear necessity for vigilant monitoring and potentially more aggressive management strategies for older Latino/Hispanic patients with melanoma, even when initial lymph node evaluations are negative. Additionally, Carlson *et al* [16] found that among patients with negative SLNB, tumour location in the head or neck and increased lesion thickness were significant predictors of regional recurrence following a negative SLNB. Notably, the 5-year survival rate for patients with regional recurrence following a negative SLNB is similar to those with positive SLNs [16]. These insights underscore the importance of considering both patient demographics and tumour characteristics in the management of melanoma, ensuring that high-risk groups receive appropriate attention and care.

When referring to distant metastasis, lymph node-negative disease is typically associated with better outcomes than lymph node-positive disease [4]. In our study, a Breslow index greater than 4 mm emerged as the sole variable linked to increased rates of distant metastasis in multivariate analysis. This underscores the critical importance of tumour thickness in risk stratification, even among patients with negative lymph nodes. It suggests that Latino/Hispanic patients with thick melanomas may benefit from more intensive surveillance and consideration for adjuvant therapies, despite negative lymph node status.

Studies by O’Connell *et al* [17] in Ireland and Lee *et al* [18] in Taiwan also found that disease recurrence was more common in cases of thicker primary melanoma, indicating that this factor is intrinsic to the disease rather than being influenced by geography or heritage. Similarly, Sun *et al* [19] identified clinical and pathological characteristics such as *N* stage, tumour size, ulceration and pathological subtype as risk predictors of distant metastasis at the time of melanoma diagnosis. The timeline of metastasis development in our cohort aligns with previous research, with distant metastases taking the longest time to develop [20]. Our average follow-up time of 25 months may have increased the likelihood of detecting distant metastasis lesions, highlighting the importance of long-term surveillance in this patient population.

Moreover, our results showed that stage IB had a tendency to metastasise to lungs, while no relation to any site in stage II, and those with more than 2 mitosis per mm^2^ had a higher frequency of metastasis to solid organs, especially the brain. This is crucial information in terms of symptoms and clinical features to look for among patients with these characteristics, which are unique to the Hispanic/Latino population with negative lymph nodes. With a high rate of recurrence of 26.9% in total, especially in stages IIB and IIC with 22% and 55% at 3-year follow, respectively, compared to previous reports in the United States with 35% and 45% at 5 years follow-up, and Europe [21–23], surveillance should be cautiously performed as patient-directed by physicians based on these findings with optimal medical decision making to assess and diagnose any metastasis in a timely manner to expedite appropriate treatment.

The best predictor of OS in lymph node-negative melanoma was a Breslow depth greater than 4 mm. This finding is consistent with our observations that patients with a Breslow thickness greater than 4 mm tended to be older, present with ulcerating lesions, experience higher recurrence rates, have more mitoses per mm^2^ and meet the criteria for AJCC stage II. These results highlight the necessity of comprehensive risk assessment in Latino/Hispanic patients, even when lymph nodes are negative. Interestingly, a study by Khosrotehrani *et al* [24] reported improved survival in females across all tumour stages of melanoma, though this was significantly influenced by age at presentation, aligning with findings from Lim *et al* [25]. Similarly, a report on conditional survival in melanoma in the Netherlands from 1994 to 2008 supported the observation that females had better survival rates, particularly when associated with low Breslow index and nodal stage [26]. In contrast, a 13-year survival analysis of melanoma in the United States indicated worse survival outcomes with increasing age and later stage at diagnosis [27].

A strength of this study is its population-specific data on lymph node-negative melanoma outcomes within Latino-Hispanic populations, contributing to the broader knowledge of prognostic factors in melanoma. However, several limitations should be noted. The retrospective design limits the ability to control for confounding factors and establish causality. As a single-center study conducted at a national referral cancer institution, the results may not be fully generalisable to other settings or populations. The possibility of false-negative SLNB must be considered when interpreting the data. On average, 13% of SLNBs are false negatives (5.6%–21%) [9]. The study’s findings should be validated in larger, multi-center prospective studies to confirm their applicability across diverse populations and clinical settings.

Our findings contribute to the understanding of prognostic factors and disease patterns in Latino/Hispanic patients with lymph node-negative melanoma. Identifying high-risk subgroups based on age and Breslow depth can inform risk stratification, surveillance strategies and potential adjuvant therapy considerations specific to this population. Further prognostic factors need to be investigated and identified in this population such as molecular and genetic markers to build new nomograms that can predict recurrence. Moreover, the advent of systemic therapies, and more specifically adjuvant immunotherapy, have questioned the utility of the SNLB in certain patient populations in resource-rich settings [28]; however, the current lack of availability of most chemotherapy agents and immunotherapy in Peru renders characterisation of prognostic factors in lymph node-negative melanoma even more important. Ongoing efforts to improve the accuracy and reliability of diagnostic tools like SLNB are crucial for optimising patient management and outcomes. Incorporating novel imaging techniques or molecular assays alongside SLNB could reduce false-negative rates and enhance staging precision.

In conclusion, this study provides valuable insights into the patterns and prognostic factors associated with recurrence in Latino/Hispanic patients with lymph node-negative melanoma. Key factors identified include older age, increased Breslow depth and number of mitosis per mm^2^. These findings emphasise the need for personalised risk assessment and management strategies in this population, potentially including more intensive surveillance and consideration for adjuvant therapies in high-risk subgroups. Further research is needed to fully elucidate the interplay between ethnicity, tumour characteristics and outcomes in melanoma, ultimately leading to improved care for all patients.

## Conflicts of interest

The authors have no conflicts of interest to disclose.

## Funding

This research received no specific grant from any funding agency in the public, commercial or not-for-profit sectors.

## Ethical considerations

This manuscript has followed the work center’s protocols for the publication of patient data and was approved for publication by the Institution’s Research Ethics Committee. Privacy has been respected, keeping the patient’s identification data confidential. The use of informed consent was not required.

## Data availability statement

The data that support the findings of this study are available from the corresponding author upon reasonable request.

## Author contributions

JJF: Conceptualisation – Ideas; data curation; methodology; validation; visualisation; writing – original draft; writing – review & editing.

JG: Conceptualisation – Ideas; data curation; methodology; validation; visualisation; writing – original draft; writing – review & editing.

GDK: Conceptualisation – Ideas; data curation; methodology; validation; visualisation; writing – original draft; writing – review & editing.

RH: visualisation; writing – original draft; writing – review & editing.

AD: visualisation; writing – original draft; writing – review & editing.

CF: visualisation; writing – original draft; writing – review & editing.

VM: visualisation; writing – original draft; writing – review & editing.

JM: visualisation; writing – original draft; writing – review & editing.

GZ: methodology; validation; visualisation; writing – original draft; writing – review & editing.

## Figures and Tables

**Figure 6.**
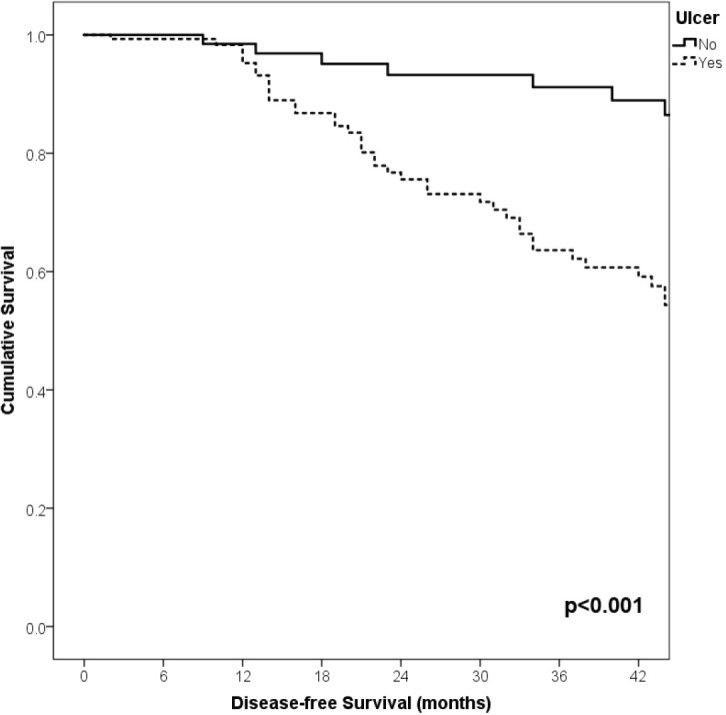
Comparison of RFS from Latino/Hispanic patients with melanoma and negative SLNs according to the presence of ulcer.

**Figure 7.**
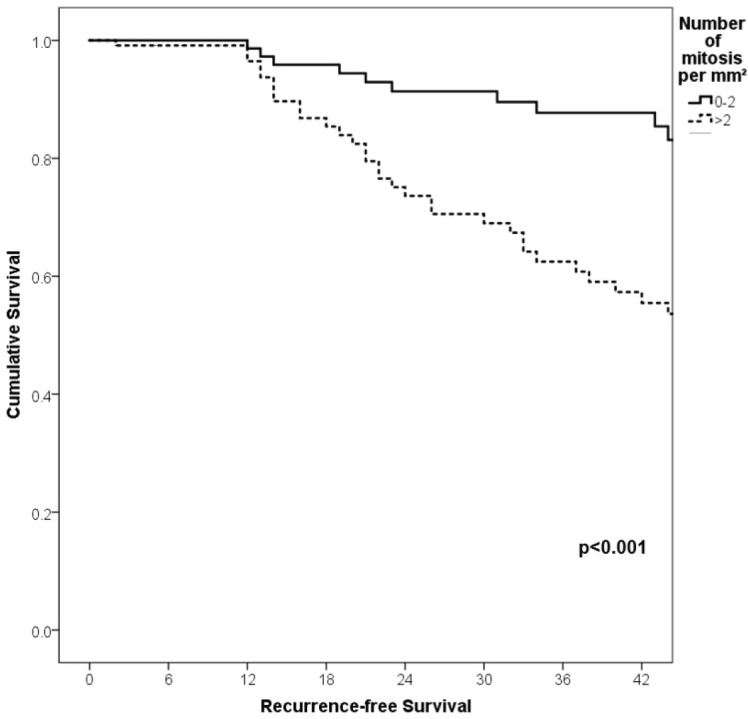
Comparison of RFS from Latino/Hispanic patients with melanoma and negative SLNs according to the number of mitosis per mm^2^.

**Figure 8.**
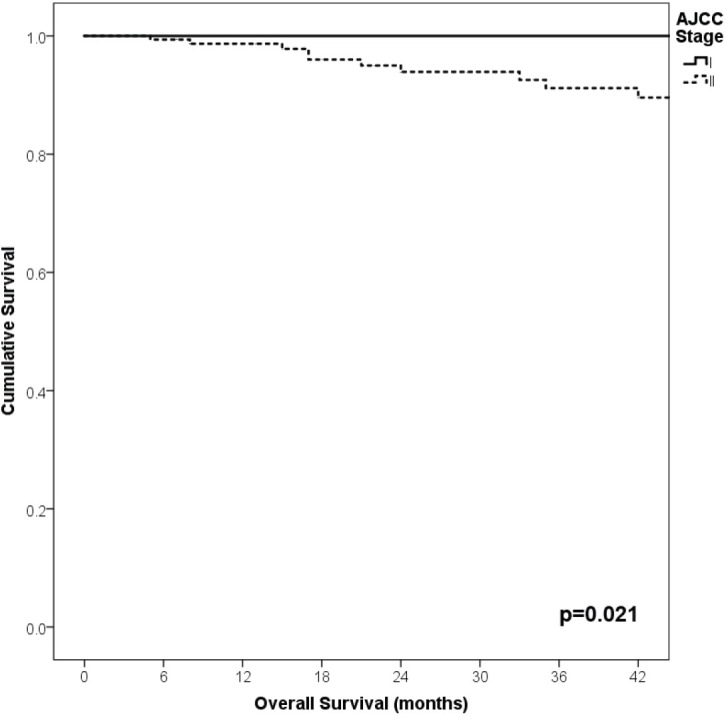
Comparison of OS from Latino/Hispanic patients with melanoma and negative SLNs according to AJCC stage.

**Figure 9.**
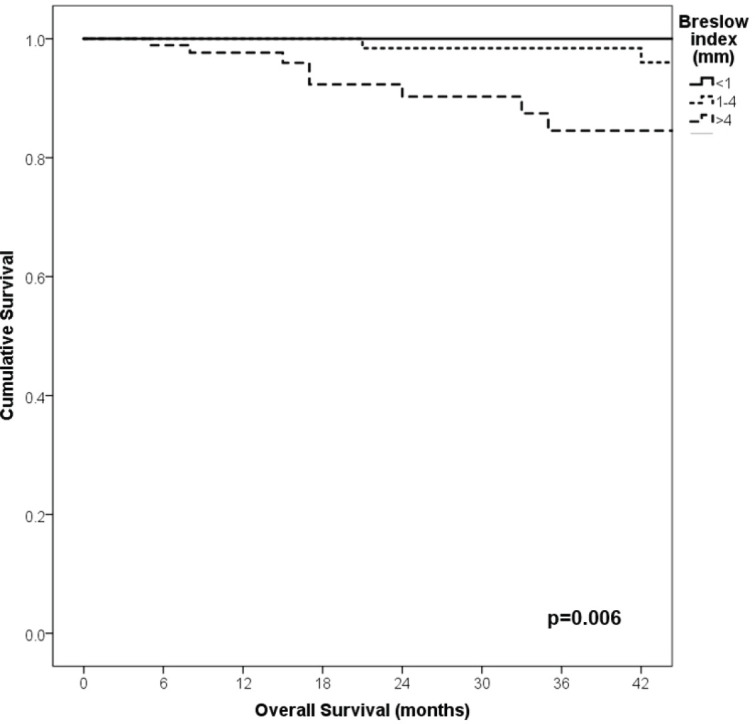
Comparison of OS from Latino/Hispanic patients with melanoma and negative SLNs according to Breslow index.

**Figure 10.**
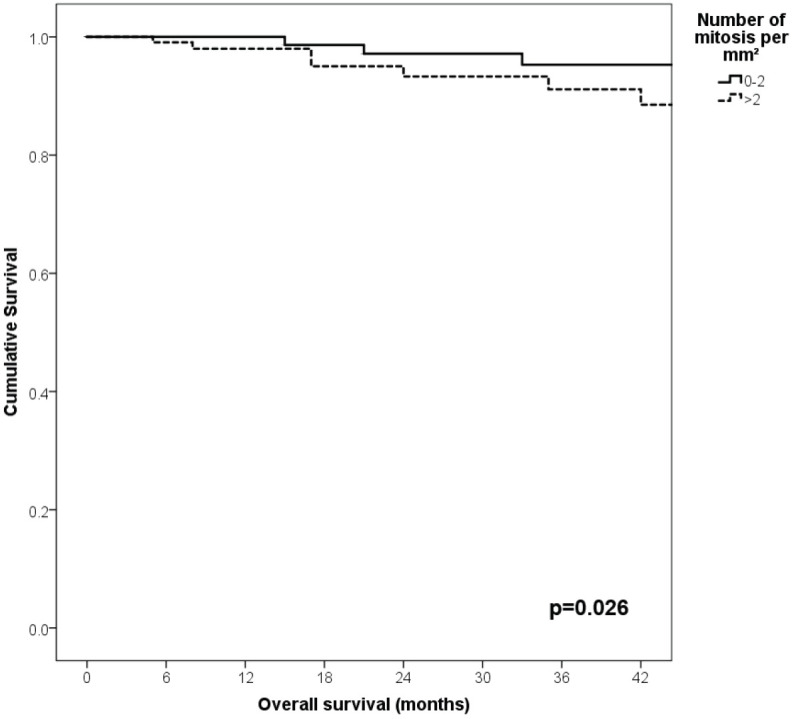
Comparison of OS from Latino/Hispanic patients with melanoma and negative SLNs according to number of mitosis per mm^2^.

**Table 1.** Sociodemographic, clinical and surgical characteristics of patients with diagnosis of melanoma according to recurrence.

Variables	Recurrence			p value
	No	Yes	Total	
Total	182	67	249	
Age, years – mean (SD)	60.64 (16.03)	65.91 (13.43)	62 (15.52)	0.017^a^
<65	97 (53.3)	22 (32.8)	119 (47.8)	0.005^b^
65–79	65 (35.7)	39 (58.2)	104 (41.8)	
≥ 80	20 (11.0)	6 (9.0)	26 (10.4)	
Sex				
Female	100 (54.9)	40 (59.7)	140 (56.2)	0.502^b^
Male	82 (45.1)	27 (40.3)	109 (43.8)	
Residence				
Metropolitan areas	99 (54.4)	32 (47.8)	131 (52.6)	0.352^b^
Rural areas	83 (45.6)	35 (52.2)	118 (47.4)	
Anatomic site				
Lower extremity	127 (72.2)	50 (74.6)	177 (72.8)	0.859^b^
Trunk	20 (11.4)	6 (9.0)	26 (10.7)	
Upper extremity	29 (16.5)	11 (16.4)	40 (16.5)	
Missing	6	0	6	
Surgery of primary lesion				
Wide local excision	100 (54.9)	47 (72.3)	147 (59.5)	0.113^c^
Other institution with re-excision of margins	22 (12.1)	5 (7.7)	27 (10.9)	
Amputation of fingers	52 (28.6)	13 (20.0)	65 (26.3)	
Extremity amputation	3 (1.6)	0 (0.0)	3 (1.2)	
Surgical biopsy without definitive surgery	5 (2.7)	0 (0.0)	5 (2.0)	
Missing	0	2	2	
Histologic subtype				
Superficial spreading	13 (7.2)	1 (1.5)	14 (5.6)	0.321 ^c^
Acral lentiginous	72 (39.8)	32 (47.8)	104 (41.9)	
Lentigo malignant	1 (0.6)	0 (0.0)	1 (0.4)	
Amelanotic	2 (1.1)	1 (1.5)	3 (1.2)	
Nodular	28 (15.5)	11 (16.4)	39 (15.7)	
Other subtypes	8 (4.4)	0 (0.0)	8 (3.2)	
NOS	58 (31.5)	22 (32.8)	80 (31.9)	
Ulcer				
No	79 (43.9)	14 (21.2)	93 (37.8)	0.001^b^
Yes	101 (56.1)	52 (78.8)	153 (62.2)	
Missing	2	1	3	
Breslow – mean (SD)	4.65 (6.13)	7.42 (6.69)	5.39 (6.39)	0.002^a^
Number of mitosis per mm^2^				
0–2	88 (52.4)	18 (28.6)	106 (45.9)	0.001^b^
>2	80 (47.6)	45 (71.4)	125 (52.1)	
Missing	14	4	18	
Total lymph nodes – mean (SD)	2.32 (1.22)	2.22 (1.13)	2.29 (1.20)	0.574^a^
Satellite lesions				
Absent	175 (98.3)	65 (98.5)	240 (98.4)	1.000^c^
Present	3 (1.7)	1 (1.5)	4 (1.6)	
Missing	4	1	5	
Regression				
No	173 (98.3)	65 (100.0)	238 (98.8)	0.566^c^
Yes	3 (1.7)	0 (0.0)	3 (1.2)	
Missing	6	2	8	
AJCC				
I	57 (31.3)	6 (9.0)	63 (25.3)	<0.001^b^
II	125 (68.7)	61 (91.0)	186 (74.7)	
Adjuvant chemotherapy				
No	175 (96.2)	57 (86.4)	232 (93.5)	0.006^b^
Yes	7 (3.8)	9 (13.6)	16 (6.5)	
Missing	0	1	1	
Adjuvant radiotherapy				
No	180 (99.4)	64 (97.0)	244 (98.8)	0.175^c^
Yes	1 (0.6)	2 (3.0)	3 (1.2)	
Missing	1	1	2	

SD, standard deviation; AJCC, American Joint Committee on Cancer

a Student-*t* test

b chi-square test

c Fisher’s exact test

**Table 2.** Patterns of metastasis of patients with diagnosis of melanoma with negative lymph nodes.

Variables		
	*N*	%
Recurrence		
Yes	67	73.1
No	182	26.9
Sites of recurrence		
Lymph nodes	21	8.4
Brain	11	4.4
Liver	4	1.6
Lung	25	10.0
Skin and subcutaneous tissue	23	9.2
Bone	6	2.4
Gastrointestinal tract	2	0.8
Multiple	21	8.4
Solid organs	32	12.9

**Table 3.** Sociodemographic, clinical and surgical characteristics of patients with diagnosis of melanoma with negative lymph nodes according to Breslow.

Variables	Breslow (mm)				p value
	≤1.00	1.01–4.00	>4.00	Total	
Total	35	109	105	249	
Age, years – mean (standard deviation)	56.97 (15.35)	60.79 (15.29)	65.08 (15.34)	62 (15.52)	0.014^a^
<65	23 (65.7)	55 (50.5)	41 (39.0)	119 (47.8)	0.021^b^
65–79	11 (31.4)	46 (42.2)	47 (44.8)	104 (41.8)	
≥80	1 (2.9)	8 (7.3)	17 (16.2)	26 (10.4)	
Sex					
Female	21 (60.0)	66 (60.6)	53 (50.5)	140 (56.2)	0.295^b^
Male	14 (40.0)	43 (39.4)	52 (49.5)	109 (43.8)	
Procedence					
Metropolitan areas	23 (65.7)	58 (53.2)	50 (47.6)	131 (52.6)	0.176^b^
Rural areas	12 (34.3)	51 (46.8)	55 (52.4)	118 (47.4)	
Anatomic site					
Lower extremity	21 (61.8)	81 (77.1)	75 (72.21)	177 (72.8)	0.474^b^
Trunk	6 (17.6)	9 (8.6)	11 (10.6)	26 (10.7)	
Upper extremity	7 (20.6)	15 (14.3)	18 (17.3)	40 (16.5)	
Missing	1	4	1	6	
Surgery of primary lesion					
Wide local excision	23 (65.7)	69 (63.3)	55 (53.4)	147 (59.5)	0.064^c^
Other institution with re-excision of margins	3 (8.6)	17 (15.6)	7 (6.8)	27 (10.9)	
Amputation of fingers	9 (25.7)	21 (19.3)	35 (34.0)	65 (26.3)	
Extremity amputation	0 (0.0)	0 (0.0)	3 (2.9)	3 (1.2)	
Surgical biopsy without definitive surgery	0 (0.0)	2 (1.8)	3 (2.9)	5 (2.0)	
Missing	0	0	1	1	
Histologic subtype					
Acral lentiginous	13 (37.1)	53 (49.1)	38 (36.2)	104 (41.9)	0.041^b^
Nodular	2 (5.7)	14 (13.0)	23 (21.9)	39 (15.7)	
Other subtypes	20 (57.1)	41 (38.0)	44 (41.9)	105 (42.3)	
Missing	0	1	0	1	
Ulcer					
No	29 (85.3)	47 (43.5)	17 (16.3)	93 (37.8)	<0.001^b^
Yes	5 (14.7)	61 (56.5)	87 (83.7)	153 (62.2)	
Missing	0	1	1	2	
Number of mitosis per mm^2^					
0–2	25 (78.1)	55 (55.0)	26 (26.3)	106 (45.9)	<0.001^b^
>2	7 (21.9)	45 (45.0)	73 (73.7)	125 (52.1)	
Missing	3	9	6	18	
Total lymph nodes – mean (SD)	2.32 (1.12)	2.17 (1.13)	2.29 (1.20)	2.29 (1.20)	0.297^a^
Satellite lesions					
Absent	34 (100.0)	107 (100.0)	99 (96.1)	240 (98.4)	0.062^c^
Present	0 (0.0)	0 (0.0)	4 (3.9)	4 (1.6)	
Missing	1	2	2	5	
Regression					
No	35 (100.0)	104 (100.0)	99 (97.1)	238 (98.8)	0.126^c^
Yes	0 (0.0)	0 (0.0)	3 (2.9)	3 (1.2)	
Missing	0	5	3	8	
AJCC					
I	35 (100.0)	27 (25.0)	0 (0.0)	63 (25.3)	<0.001^b^
II	0 (0.0)	82 (75.0)	102 (100.0)	186 (74.7)	
Adjuvant chemotherapy					
No	35 (100.0)	101 (92.7)	96 (92.3)	232 (93.5)	0.244^b^
Yes	0 (0.0)	8 (7.3)	8 (7.7)	16 (6.5)	
Missing	0	0	1	1	
Adjuvant radiotherapy					
No	35 (100.0)	107 (98.2)	102 (99.0)	244 (98.8)	0.660^c^
Yes	0 (0.0)	2 (1.8)	1 (1.0)	3 (1.2)	
Missing	0	0	2	2	
Recurrence					
No	35 (100.0)	78 (71.6)	69 (65.7)	182 (73.1)	<0.001^b^
Yes	0 (0.0)	31 (28.4)	36 (34.3)	67 (26.9)	
Lymph node recurrence					
No	35 (100.0)	96 (88.1)	97 (92.4)	228 (91.6)	0.081^b^
Yes	0 (0.0)	13 (11.9)	8 (7.6)	21 (8.4)	
Distant metastasis					
No	34 (97.1)	94 (86.2)	86 (81.9)	214 (85.9)	0.080^b^
Yes	1 (2.9)	15 (13.8)	19 (18.1)	35 (14.1)	
Multiple					
No	35 (100)	101 (92.7)	92 (87.6)	228 (91.6)	0.064^b^
Yes	0 (0)	8 (7.3)	13 (12.4)	21 (8.4)	
Solid organs					
No	34 (97.1)	95 (87.2)	88 (83.8)	217 (97.1)	0.125^b^
Yes	1 (2.9)	14 (12.8)	17 (16.3)	32 (12.9)	
Death					
No	35 (100.0)	103 (94.5)	93 (88.6)	231 (92.8)	0.049^b^
Yes	0 (0.0)	6 (5.5)	12 (11.4)	18 (7.2)	

SD, standard deviation; AJCC, American Joint Committee on Cancer

a Student-*t* test

b chi-square test

c Fisher’s exact test

**Table 4.** Site of metastasis of patients with diagnosis of melanoma with negative lymph nodes according to pathological variables.

Variables	Site of metastasis									
	Lymph nodes *N* = 21	*p* value	Lung *N* = 25	*p* value	Brain *N* = 11	*p* value	Multiple *N* = 21	*p* value	Solid organs *N* = 32	*p* value
AJCC stage										
I	2 (9.5)	0.082^a^	3 (12.0)	0.107^a^	1 (9.1)	0.206^a^	1 (4.8)	0.024^a^	4 (12.5)	0.074^a^
II	19 (90.5)		22 (88.0)		10 (90.9)		20 (95.2)		28 (87.5)	
Only stage I										
IA	0 (0.0)	0.141^b^	0 (0.0)	0.051^b^	0 (0.0)	0.648^b^	0 (0.0)	0.381^b^	0 (0.0)	0.150^b^
IB	2 (100.0)		3 (100.0)		0 (0.0)		1 (100.0)		3 (100.0)	
Only stage II										
IIA	4 (21.0)	0.690^a^	4 (18.2)	0.390^a^	1 (10.0)	0.167^b^	4 (20.0)	0.107^b^	4 (14.3)	0.316^a^
IIB	7 (36.8)		4 (18.2)		1 (10.0)		2 (10.0)		6 (21.4)	
IIC	8 (42.1)		14 (63.6)		8 (80.0)		14 (70.0)		18 (64.3)	
Ulcer										
No	5 (23.8)	0.167^a^	7 (29.2)	0.358^a^	3 (27.3)	0.461^a^	6 (28.6)	0.362^a^	8 (25.8)	0.141^a^
Yes	16 (76.2)		17 (70.8)		8 (72.7)		15 (71.4)		23 (74.2)	
Missing	0		1		0		0		1	
Number of mitosis per mm^2^										
0–2	7 (36.8)	0.409^a^	8 (33.3)	0.192^a^	2 (18.2)	0.059^a^	6 (28.6)	0.095^a^	9 (29.0)	0.043^a^
>2	12 (63.2)		16 (66.7)		9 (81.8)		15 (71.4)		22 (71.0)	
Missing	2		1		0		0		1	

AJCC, American Joint Committee on Cancer

a chi-square test

b Fisher’s exact test

**Table 5.** Univariate and multivariate cox regression analysis for RFS in Latino patients with melanoma diagnosis and negative lymph nodes.

Variables	RFS					
	Univariate analysis			Multivariate analysis		
	HR	95%CI	*p* value	HR	95%CI	*p* value
Age, years	1.024	1.005–1.042	0.011	1.017	0.997–1.038	0.095
Sex						
Female	1.000			1.000		
Male	1.113	0.681–1.818	0.670	0.728	0.395–1.339	0.307
Living						
Metropolitan areas	1.000			1.000		
Non-metropolitan areas	1.491	0.021–2.414	0.104	1.307	0.770–2.216	0.321
Anatomic site						
Lower extremity	1.000			1.000		
Trunk	0.877	0.375–2.051	0.763	2.281	0.796–6.539	0.125
Upper extremity	1.021	0.530–1.967	0.950	0.634	0.295–1.365	0.244
Ulcer						
No	1.000			1.000		
Yes	3.122	1.725–5.650	<0.001	1.968	0.974–3.978	0.059
Breslow -mm	1.114	1.079–1.151	<0.001	1.098	1.051–1.146	<0.001
Number of mitosis per mm^2^						
0–2	1.000			1.000		
>2	2.992	1.729–5.177	<0.001	2.105	1.150–3.852	0.016

**Table 6.** Univariate and multivariate cox regression analysis for lymph node recurrence in Latino patients with melanoma diagnosis and negative lymph nodes.

Variables	Lymph node recurrence						
	Univariate analysis				Multivariate analysis		
	HR	95%CI	*p* value	HR		95%CI	*p* value
Age, years	1.045	1.008–1.084	0.016	1.053		1.010–1.098	0.016
Sex							
Female	1.000			1.000			
Male	1.232	0.517–2.938	0.638	1.122		0.364–2.458	0.842
Living							
Metropolitan areas	1.000			1.000			
Non-metropolitan areas	1.844	0.773–4.399	0.168	2.397		0.842–6.823	0.101
Anatomic site							
Lower extremity	1.000			1.000			
Trunk	1.000	0.228–4.391	1.000	3.472		0.535–12.551	0.192
Upper extremity	1.257	0.416–3.798	0.685	1.299		0.375–4.504	0.680
Ulcer							
No	1.000			1.000			
Yes	2.612	0.951–7.173	0.063	3.224		0.872–11.917	0.079
Breslow -mm	1.045	0.967–1.128	0.264	0.967		0.859–1.089	0.585
Number of mitosis per mm^2^							
0–2	1.000			1.000			
>2	2.022	0.794–5.147	0.140	1.268		0.447–3.597	0.655

**Table 7.** Univariate and multivariate cox regression analysis for distant metastasis in Latino patients with melanoma diagnosis and negative lymph nodes.

Variables	Distant metastasis free survival						
	Univariate analysis				Multivariate analysis		
	HR	95%CI	*p* value	HR		95%CI	*p* value
Age, years	1.012	0.989–1.036	0.305	1.009		0.983–1.036	0.508
Sex							
Female	1.000			1.000			
Male	1.066	0.541–2.099	0.854	0.493		0.208–1.167	0.108
Living							
Metropolitan areas	1.000			1.00			
Non-metropolitan areas	1.393	0.717–2.705	0.328	1.123		0.552–2.285	0.749
Anatomic site							
Lower extremity	1.00			1.00			
Trunk	1.530	0.583–4.015	0.387	3.988		0.88–11.453	0.114
Upper extremity	1.226	0.501–3.002	0.656	0.930		0.349–2.478	0.885
Ulcer							
No	1.000			1.000			
Yes	2.351	1.094–5.051	0.028	1.267		0.527–3.046	0.598
Breslow -mm	1.128	1.074–1.185	<0.001	1.126		1.059–1.196	<0.001
Number of mitosis per mm^2^							
0–2	1.000			1.000			
>2	2.499	1.216–5.135	0.013	2.021		0.900–4.536	0.088

**Table 8.** Univariate and multivariate cox regression analysis for OS in Latino patients with melanoma diagnosis and negative lymph nodes.

Variables	OS					
	Univariate analysis			Multivariate analysis		
	HR	95%CI	*p* value	HR	95%CI	*p* value
Age, years	1.012	0.979–1.046	0.478	1.002	0.965–1.031	0.884
Sex						
Female	1.000			1.000		
Male	0.821	0.307–2.194	0.694	0.873	0.294–2.588	0.806
Living						
Metropolitan areas	1.000			1.000		
Non-metropolitan areas	0.682	0.255–1.822	0.445	0.650	0.232–1.821	0.412
Anatomic site						
Lower extremity	1.000			1.000		
Trunk	0.475	0.063–3.583	0.470	0.762	0.080–7.251	0.813
Upper extremity	0.301	0.040–2.275	0.245	0.298	0.039–2.281	0.244
Ulcer						
No	1.000			1.000		
Yes	2.888	0.947–8.809	0.062	1.420	0.409–4.936	0.581
Breslow -mm	1.111	1.063–1.162	<0.001	1.090	1.034–1.150	0.001
Number of mitosis per mm^2^						
0–2	1.000			1.000		
>2	3.058	1.088–8.594	0.034	1.876	0.589–5.977	0.287

**Table 9.** RFS and OS rates of patients with triple-negative breast cancer according to pathological stage.

	RFS				OS			
Results	1 year (%)	2 years (%)	3 years (%)	*p* value	1 year (%)	2 years (%)	3 years (%)	*p* value
All population	97	83	75		99	96	94	
Age (years)								
<65	97	85	81	0.015	100	99	97	0.356
65–79	96	79	66		100	93	91	
80+	100	90	90		89	89	89	
Histologic subtype								
Acral lentiginous	98	83	73	0.696	100	97	95	0.459
Nodular	91	73	73		100	95	95	
Other subtypes	96	85	77		97	94	91	
AJCC stage								
I	100	100	94	<0.001	100	100	100	0.021
II	96	77	68		99	94	91	
Stage I								
IA	100	100	100	<0.001	100	100	100	0.046
IB	100	100	84		100	100	67	
Stage II								
IIA	100	84	74	0.005	100	100	100	0.109
IIB	97	85	78		100	97	93	
IIC	92	66	58		97	89	86	
Breslow index								
<1 mm	100	100	100	<0.001	100	100	100	0.006
1–4 mm	99	89	75		100	98	98	
>4 mm	96	69	64		98	90	85	
Ulcer								
No	98	93	91	<0.001	100	100	98	0.051
Yes	95	76	64		98	93	91	
Number of mitosis per mm^2^								
0–2	98	91	88	<0.001	100	97	95	0.026
>2	97	74	63		98	93	91	

AJCC, American Joint Committee on Cancer
